# Increasing C-reactive protein levels in a patient with glioblastoma with lymph node metastasis: a case report

**DOI:** 10.1186/s12883-023-03402-4

**Published:** 2023-10-04

**Authors:** Takuya Kanemitsu, Motomasa Furuse, Hiroko Kuwabara, Ryokichi Yagi, Ryo Hiramatsu, Masahiro Kameda, Naosuke Nonoguchi, Shinji Kawabata, Toshihiro Takami, Motohiro Arai, Masahiko Wanibuchi

**Affiliations:** 1 Department of Neurosurgery, Osaka Medical and Pharmaceutical University, Takatsuki, Osaka Japan; 2 Department of Neurosurgery, Midorigaoka Hospital, Takatsuki, Osaka Japan; 3 Department of Pathology, Osaka Medical and Pharmaceutical University, Takatsuki, Osaka Japan

**Keywords:** C-reactive protein, Extracranial metastasis, Glioblastoma, Lymph node metastasis

## Abstract

**Background:**

Glioblastoma usually recurs locally and extracranial metastases are rare. Most patients with extracranial metastases experience recurrence of the primary intracranial tumor. Lymph node metastases are often detected based on lymphadenopathy or symptoms caused by other metastatic sites.

**Case presentation:**

Herein, we report a case of glioblastoma with lymph node metastasis in which the patient was asymptomatic but exhibited gradually increasing C-reactive protein levels prior to becoming febrile 9 months after the initial C-reactive protein increase. Diagnosis of lymph node metastasis that was delayed because the patient had a fever of unknown origin, no signs of infection, and the primary intracranial tumor did not recur. Chest computed tomography indicated supraclavicular, mediastinal, and hilar lymphadenopathy, and biopsy identified lymph node metastasis of glioblastoma. This is the fifth reported case of lymph node metastasis without intracranial recurrence.

**Conclusions:**

C-reactive protein levels may be a diagnostic marker for lymph node metastasis in patients with glioblastoma. Further evaluation is needed to elucidate the role of CRP in glioblastoma with lymph node metastasis.

## Background

Glioblastoma (GBM) has a high recurrence rate and poor prognosis [1]. GBM often recurs locally around the initial lesion, and extracranial metastases are extremely rare [2–5]. The reported frequency of extracranial metastases is 0.2–2% [6], with metastases to the lung/pleura (60%), lymph nodes (51%), bone (31%), liver (22%), scalp, kidney, orbit, spleen, and heart [7–10]. In most cases, recurrence of intracranial tumors was also present. Although the pathogenesis of metastasis remains unclear, Piccirilli et al. reported that extracranial metastasis occurred after 16–23 months and the outcome was poor despite good progress after surgery and adjuvant chemoradiotherapy [10].

Here, we present a case of GBM with lymph node metastases without intracranial recurrence. Lymph node metastasis detection was delayed because the patient did not exhibit any symptoms other than low-grade fever. However, C-reactive protein (CRP) levels had gradually increased until metastasis was diagnosed. Therefore, we retrospectively assume that CRP may be a marker for lymph node metastasis in patients with glioblastoma.

## Case presentation

**Fig. 1:**
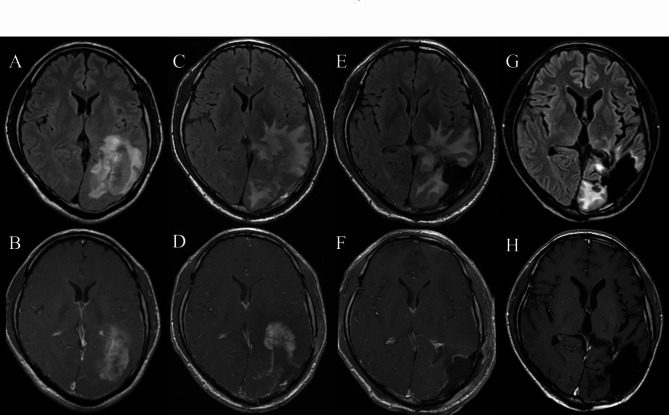
Summary of magnetic resonance imaging axial images. Preoperative magnetic resonance imaging (MRI) reveals a heterogeneously gadolinium-enhanced lesion with edema in the left temporo-occipital lobe (**A**: FLAIR, **B**: contrast-enhanced T1). MRI before the second surgery shows a contrast-enhanced lesion with edema deep in the postoperative cavity (**C**: FLAIR, **D**: contrast-enhanced T1). MRI after the second surgery shows that all contrast-enhanced lesions have been resected (**E**: FLAIR, **F**: contrast-enhanced T1). The final MRI, performed 37 months after the initial surgery, shows no intracranial tumors (**G**: FLAIR, **H**: contrast-enhanced T1)

**Fig. 2:**
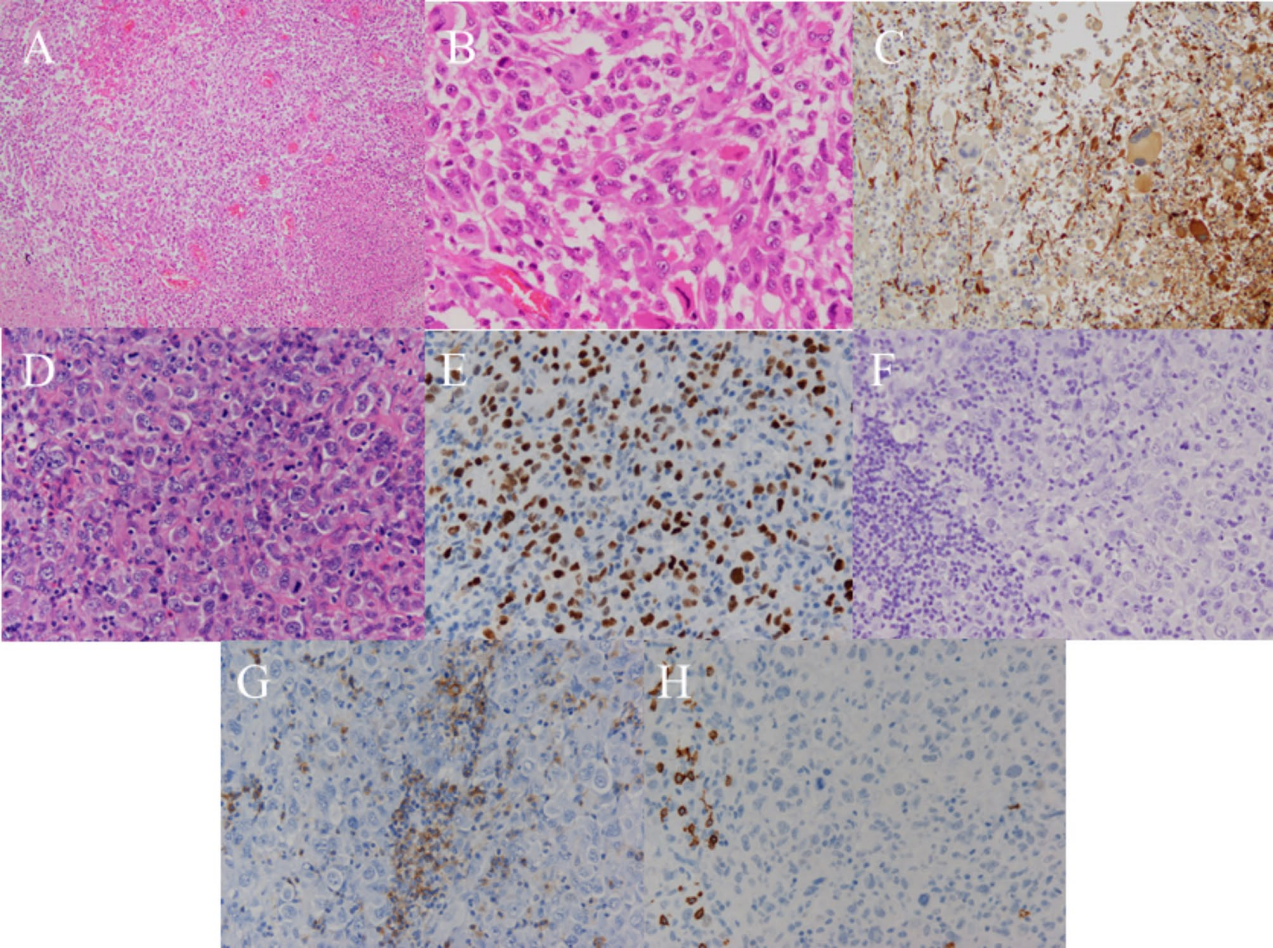
Histopathological results. Histopathological findings of the (**A**–**B**) brain tumor and (**D**–**F**) lymph nodes. Hematoxylin and eosin (HE) staining shows highly atypical tumor cells with areas of necrosis and microvascular proliferation (**A**: ×100, **B**: ×400). GFAP immunostaining is slightly positive in larger tumor cells, but negative in most cells (**C**: ×200). HE staining reveals cells with distinct nucleoli, nodding nuclei, and abundant eosinophilic endoplasmic reticulum, similar to brain tumor cells (**D**: ×400). The immunostaining results are (**E**) p53 positive, (**F**) GFAP-negative, (**G**) LCA-negative, and (**H**) CD20 negative (×200)

A 48-year-old male patient presented with dyslexia, agraphia, and homonymous hemianopia. Post-contrast magnetic resonance imaging (MRI) showed a heterogeneously enhanced lesion in the left temporo-occipital lobe (Fig. 1A, B). The tumor was subtotally resected and carmustine wafers were implanted into the surgical cavity. The pathological diagnosis was GBM, IDH-wildtype (Fig. 2A, B, C). Radiotherapy (60 Gy in 30 fractions) with concomitant temozolomide therapy (75 mg/m^2^) was performed after surgery [1]. One month after completion of chemoradiotherapy, wound infection was observed. The wound was debrided and treated with meropenem antibiotic therapy .

Three months after completion of chemoradiotherapy, MRI revealed an increase in the size of the contrast-enhanced tumor. A second craniotomy was therefore performed (Fig. 1C, D) and the lesions were completely resected (Fig. 1E, F). Thereafter, the patient was treated with adjuvant temozolomide (150 mg/m^2^) and bevacizumab (10 mg/kg) every 4 weeks. Follow-up included laboratory assessments every 4 weeks and MRI every 2 months. The patient had a febrile episode 2 months after the second surgery. Blood tests revealed mildly elevated CRP (1.17 mg/dL) and white blood cell (WBC) count (8760/µL). Sputum, urine, and blood cultures performed to identify the source of fever were negative, and full-body computed tomography (CT) revealed no source of the fever. There were no signs of wound infection, but there was a subcutaneous effusion, which was punctured, and culture tests detected *Serratia marcescens*. Since this bacterium was previously found in the postoperative wound infections, the patient was treated with meropenem for 2 weeks, followed by cefepime for another 2 weeks, after which the fever resolved. The patient received prophylactic trimethoprim-sulfamethoxazole and temozolomide maintenance therapy.

**Fig. 3:**
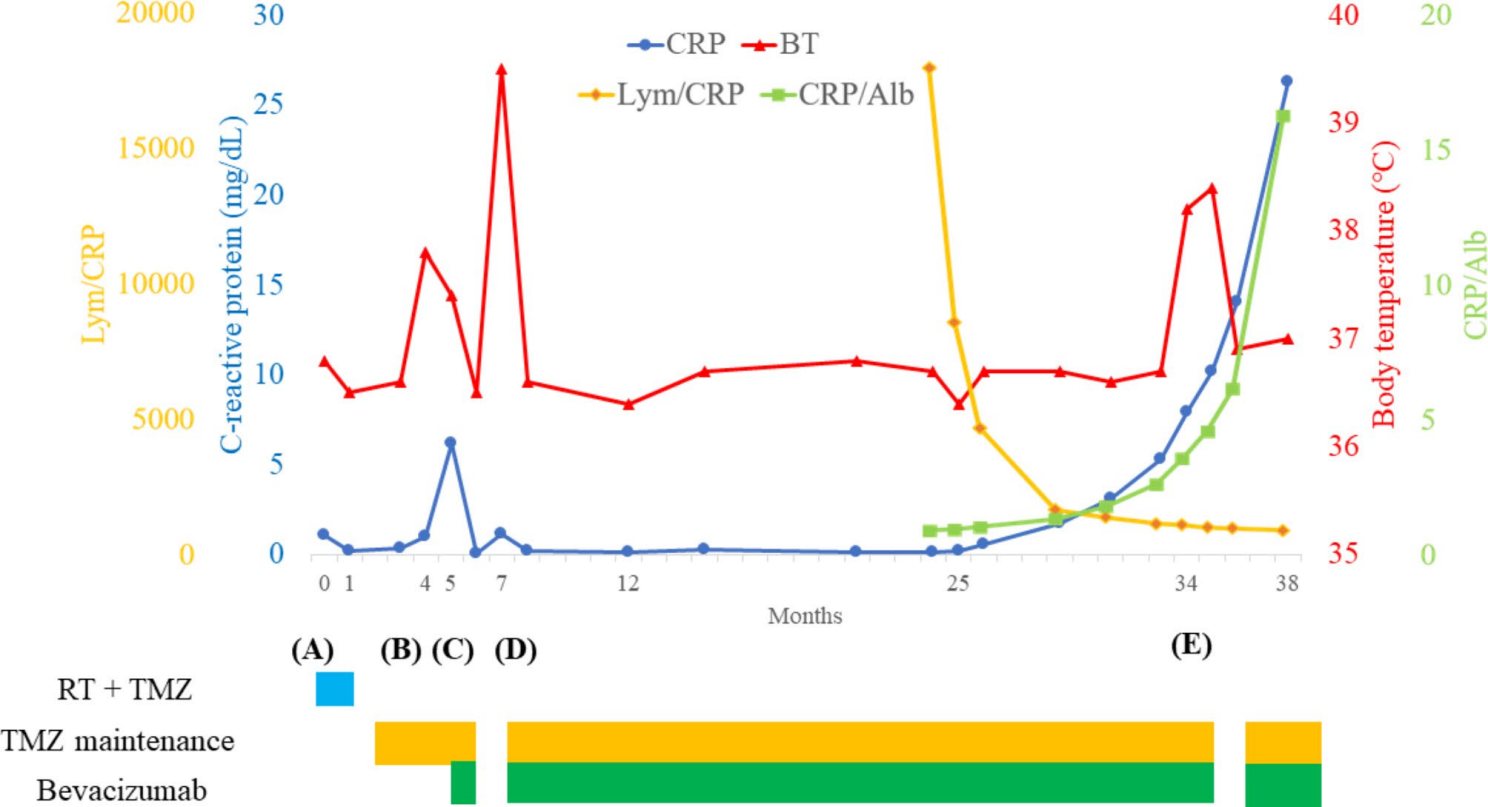
The chronological clinical course of the patient (**A**) The initial surgery is performed. (**B**) Wound infection is observed, the wound is debrided, and antibiotic therapy administered. (**C**) The patient undergoes the second surgery for recurrence after 5 months. (**D**) The patient receives antibiotic treatment for fever of unknown origin. (**E**) Chest computed tomography, performed due to high-grade fever, shows lymphadenopathy and a lymph node biopsy is performed. Lym/CRP: lymphocyte-CRP ratio, CRP/Alb: CRP-albumin ratio, RT: radiotherapy, TMZ: temozolomide

**Fig. 4:**
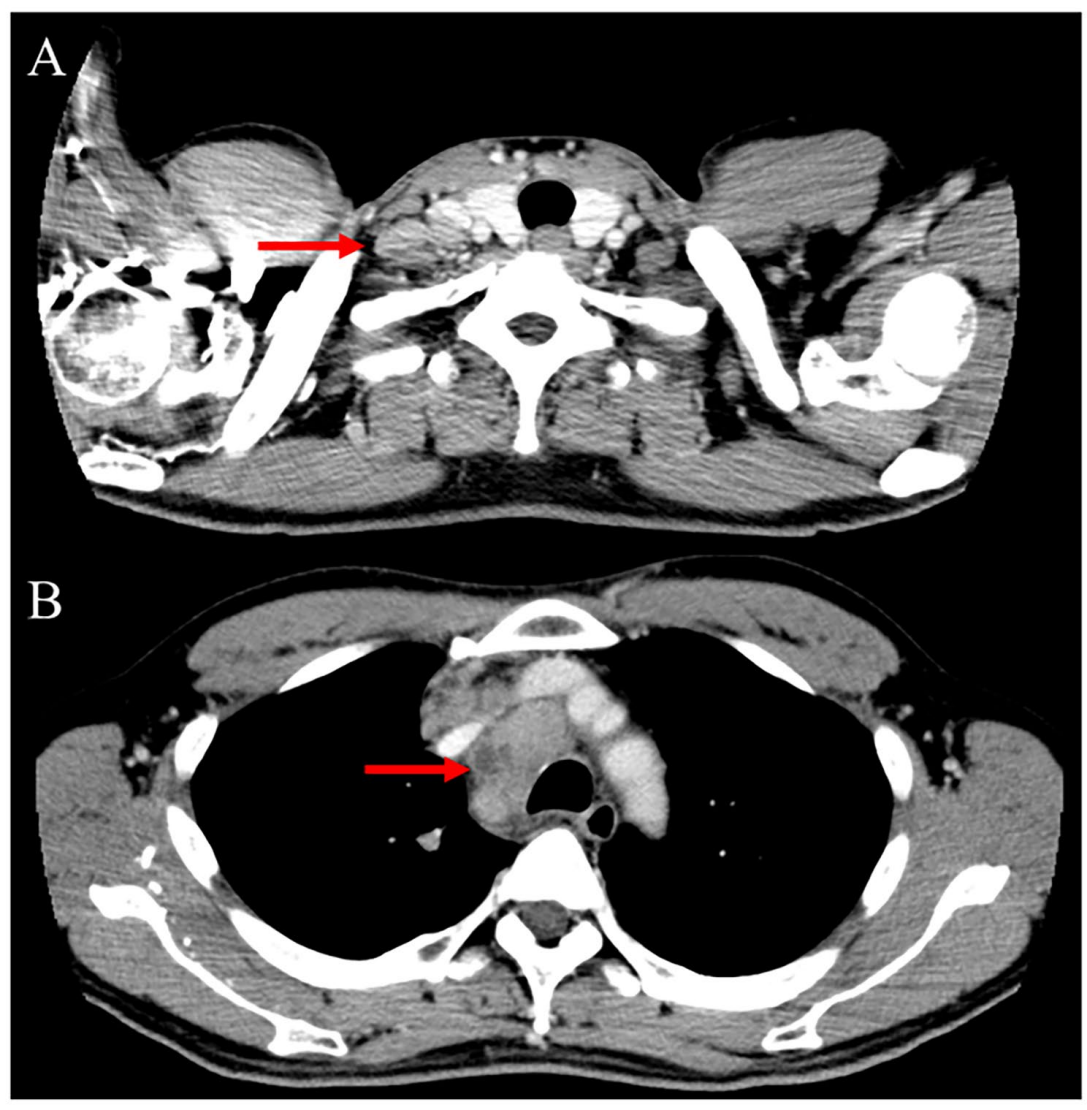
Chest computed tomography. Contrast-enhanced computed tomography showing glioblastoma metastatic lesions in the (**A**, arrow) right supraclavicular lymph node and (**B**, arrow) mediastinal lymph node

The patient’s chronological clinical course is shown in Fig. 3. An increase in CRP level was identified 20 months after the second surgery and continued to increase slowly without a concurrent increase in WBC count. Twenty-nine months after surgery, CRP was 5.29 mg/dL and body temperature was 38℃. Simultaneously, CRP-albumin ratio increased and lymphocyte–CRP ratio decreased. However, there were no signs of infection of the surgical wound or elsewhere. Chest CT performed to investigate the source of the fever revealed swelling of the supraclavicular, mediastinal, and hilar lymph nodes (Fig. 4) and lymph node biopsy was performed. We suspected secondary lymphoma associated with temozolomide because the patient had received temozolomide treatment for more than 2 years (28 therapy cycles) and serum soluble interleukin-2 receptor was elevated to 1031 U/mL. Despite the prolonged temozolomide therapy and the associated risk, maintenance temozolomide therapy was continued based on the patient’s request. However, the pathological findings were negative for lymphoma, and the patient was diagnosed with lymph node metastasis of glioblastoma (Fig. 2D–H). Lymph node metastasis progressed to the lungs, and the patient received palliative irradiation (20 Gy in 4 fractions) to the mediastinal lymph nodes. The patient died 4 months after diagnosis of metastasis (38 months after surgery) due to respiratory failure and pneumonia. The intracranial tumor had not recurred by the final follow-up MRI performed 37 months after the initial surgery (Fig. 1G, H).

## Discussion

GBM has a very poor prognosis with a median survival time of 14.6 months [1]. Extracranial metastases are rare because of the closed anatomical features of the intracranial region, including no lymphatic vessels within the central nervous system, no communication between the intracranial perivascular spaces and the extracranial fluid space, sparse connections between the subarachnoid space and extracranial lymphatic vessels, and embedded intracerebral veins, surrounded.

by astrocytes. Furthermore, the tumor does not penetrate veins as some veins collapse when the tumor approaches, and the dural veins are protected by dense connective tissue [8, 11]. Five explanations for extracranial metastases in GBM have been proposed [8], including hematogenous spread via primary tumor vessels; hematogenous spread following tumor invasion of the dural veins; hematogenous and/or lymphatic spread following infiltration of the skull and extracranial soft tissues; spread via the cerebrospinal fluid; or spread via ventriculoperitoneal or ventricular-atrial shunt. A combination of these mechanisms is also possible.

Table 1 summarizes reported cases of GBM with lymph node metastasis [5, 10, 12–26]. The cervical lymph node is the most common metastatic site, with other sites including the mediastinal, submandibular, and supraclavicular lymph nodes. Local intracranial tumor progression was observed in 12 of the 19 cases, and in most of these cases lymph node metastasis was preceded by lymphadenopathy. In five cases, including our case, lymph node metastasis was observed without intracranial progression. Piccirilli et al. [10] reported that metastasis preceded recurrence of the primary tumor in all cases; however, in all cases, an increase in intracranial lesions led to death. Blume et al. [5] reported that, even when no intracranial recurrence was observed, metastases to the vertebral body, spinal canal, and lungs result in death. Almost all patients with extracranial metastases had undergone surgery [27], and most patients with lymph node metastases had undergone several craniotomies. Lymph node metastasis is thought to occur due to surgical destruction of brain anatomical structures, allowing tumor cells to gain access to meningeal and/or scalp lymphatic vessels [8, 10, 28, 29]. However, there have also been reports of preoperative lymph node or distant metastases [29]. Because these cases showed no clinical or radiological evidence of scalp or skull transgression, tumor cell characteristics, such as particular molecular features, p53 gene mutations, and differential clonal selection, may dictate metastatic predisposition [3, 10, 25, 30]. There are various treatments for metastatic lesions, including surgery, radiation therapy, and different chemotherapy protocols. As seen in Table 1, the mean survival times for patients with and without intracranial progression were 5.6 ± 2.2 and 9.2 ± 4.2 months, respectively, with median survival times of 2.5 [1–17] and 5 [2–25] months (*p* = 0.30, log-rank test). Intracranial progression was not associated with survival time.

**Table 1:** Summary of reported glioblastoma cases with lymph node metastasis

Author	Age	Sex	Lymph node metastasis	The other metastasis	Intracranial tumor	Symptom	Duration from initial Dx	Treatment	Survival time (months)
Nigogosyan [12], 1962	40	M	Mediastinal	Lung, vertebrae, sternum	Local progression	None (autopsy)	7.5	None	N/A
Komatsu [13], 1972	18	F	Cervical	Scalp	Local progression	Lymphadenopathy	7	Surgery, radiotherapy	2
Hulbanni [14], 1976	63	M	Bronchial	Lung, vertebrae	Local progression	None (autopsy)	0	None	0
Pasquier [15], 1980	21	F	Submandibular	Liver	Local progression	Lymphadenopathy	0.5	Surgery	1
Steinbok [16], 1985	27	F	Submental and cervical	None	Local progression	Lymphadenopathy	45	Surgery, radiotherapy, chemotherapy	17
Trattnig [17], 1990	29	M	Cervical	Bones	Local progression	Retromandibular swelling	18	Surgery, radiotherapy, chemotherapy	14
Zappia [18], 1992	39	M	Submandibular and deep cervical, parotid chains	None	Local progression	Lymphadenopathy	19	Chemotherapy	N/A
Wallase [19], 1996	41	M	Cervical and supraclavicular	Extraocular muscle	None	Painful lymphadenopathy	3	Radiotherapy, chemotherapy	5
Wallase [19], 1996	37	M	Auricular	Orbit	Local progression	Headache, drowsiness, facial numbness	5	Second craniotomy	2
al-Rikabi [20], 1997	4	M	Cervical	None	N/A	Lymphadenopathy, fever	1	N/A	N/A
Montagne [21], 2004	74	F	Mediastinal	Lung, vertebrae	Local progression	Thrombocytopenia, diffuse myelalgia	0	N/A	2
Moon [22], 2004	35	F	Cervical	Scalp	Local progression	Lymphadenopathy, headache, nausea	48	Chemotherapy	4
Taha [23], 2005	33	M	Cervical, parotid gland	None	Local progression	Lymphadenopathy	5.5	Radiotherapy, chemotherapy	3
Didelot [24], 2006	74	M	Mediastinal	Bone marrow, lung	None	Pancytopenia	0	Chemotherapy	2
Piccirilli [10], 2007	70	F	Cervical	None	Local progression	Lymphadenopathy	20	N/A	10
Mujtaba[25], 2013	20	M	Supraclavicular	None	N/A	Lymphadenopathy , cough	19	Surgery, radiotherapy, chemotherapy	N/A
Blume [5], 2013	35	M	Pulmonary	Lung, vertebrae	None	Tetraparesis	N/A	Surgery, radiotherapy, chemotherapy	10
Xu [26], 2016	58	F	Cervical	Bones	None	Lymphadenopathy	29	Surgery, radiotherapy, chemotherapy	25
Kanemitsu, 2023	48	M	Supraclavicular and mediastinal	Lung	None	Fever, CRP increasing	34	Radiotherapy, chemotherapy	4

F: Female, M: male, N/A: not available

In our case, a gradual increase in CRP level was observed over 9 months, but there were no apparent symptoms. Chest CT performed to detect the cause of fever revealed swelling of the supraclavicular lymph nodes for the first time. At this time, the patient had received adjuvant temozolomide for more than 2 years and exhibited elevated soluble interleukin-2 receptor. We therefore initially suspected secondary leukemia associated with long-term temozolomide treatment [31] rather than metastatic GBM in the mediastinal lymph nodes. Although extracranial metastases in GBM have a poor prognosis, radical resection, radiotherapy, and chemotherapy are recommended as soon as possible [25].

Metastasis in the cervical lymph nodes can be detected in the early stages when it is preceded by lymphadenopathy. However, in patients with supraclavicular or mediastinal lymph node metastasis, diagnosis is delayed because of few apparent symptoms. In our case, good control of the primary tumor achieved with chemotherapy and the past history of wound infection led us to suspect latent infection as the cause of increased CRP, thus delaying detection of lymph node metastases. We retrospectively suggest that, in the absence of any signs of infection, increased CRP may indicate lymph node metastasis, even if the primary tumor is well controlled. CRP is not only an acute phase protein, but also a marker for chronic microinflammation. Cancers are usually associated with chronic inflammation and CRP is a prognostic marker for some cancers. Inflammation is associated with tumor proliferation, invasion, and metastasis, and proinflammatory cytokines contribute to formation of the tumor microenvironment for metastasis [32]. In GBM, preoperative CRP is an outcome predictor [33]. However, there are no reports of CRP associated with metastasis in GBM. Furthermore, lymphocyte–CRP and CRP–albumin ratios are prognostic predictors for many cancers [34] [35]. There are, however, no reports regarding these parameters in GBM. In our case, lymphocyte–CRP and CRP–albumin ratios also changed (Fig. 3) and may therefore also predict lymph node metastasis in GBM. Further evaluation of CRP and lymphocyte–CRP and CRP–albumin ratios is required to elucidate the correlation between these parameters and prognosis in GBM.

## Conclusions

Lymph node metastasis in GBM is rare and is usually diagnosed following lymphadenopathy. In most patients, the primary intracranial tumor recurs along with lymph node metastasis. The diagnosis of lymph node metastasis was delayed in our case because the patient became febrile without any other symptoms, and the intracranial tumor had not recurred. We retrospectively suggest that persistently increasing CRP levels may be a diagnostic marker for lymph node metastasis in patients with GBM. However, further cases are required to verify the prognostic ability of CRP in GBM with lymph node metastasis.

## Data Availability

Data and materials are available from the corresponding author on reasonable request.

## Abbreviations

CRP: RC-reactive protein
CT: Computed tomography
GBM: Glioblastoma
MRI: Magnetic resonance imaging
WBC: White blood cell
